# Retroperitoneal hematoma secondary to rupture of uterine artery pseudoaneurysm after cesarean section, managed by selective embolization: A case report

**DOI:** 10.1016/j.radcr.2026.01.039

**Published:** 2026-02-19

**Authors:** Ammar Maireche, Camelia Hamadache, Mohamed Amine Bordja, Smail Oumaamar, Wafia Boulahdour, Elmouiz Lidine Allah Maireche, Medjda Bouras, Imene Riougui, Chaffa Aimeur

**Affiliations:** a University of Algiers 1, Benyoucef Benkhedda, 2 Rue Didouche Mourad, Algiers 16000, Algeria; b Mustapha University Hospital Center, Place du 1er Mai, Sidi M’Hamed, Algiers 16000, Algeria; c Salim Zemirli Hospital, Route de Baraki, El Harrach, Algiers 16200, Algeria

**Keywords:** Uterine artery pseudoaneurysm, Secondary postpartum hemorrhage, Color Doppler ultrasound, CT angiography, Uterine artery embolization, Interventional radiology

## Abstract

A 35-year-old woman underwent a cesarean section at our hospital. Five days postoperatively, she presented with abdominal pain, vomiting, and deterioration of her general condition. Laboratory tests revealed hypotension with a blood pressure of 90/70 mmHg and a hemoglobin level of 6 g/dL, without any signs of external bleeding. After initial stabilization, an ultrasound examination with Doppler followed by a CT scan was performed, demonstrating a retroperitoneal hematoma and a saccular vascular lesion of the uterine artery consistent with a pseudoaneurysm. The patient was successfully treated by selective arterial embolization, resulting in excellent clinical outcomes. This case illustrates the importance of Doppler ultrasound in establishing a prompt diagnosis, particularly for nonexperienced radiologists, and highlights selective embolization as an effective and minimally invasive treatment option for postpartum hemorrhage caused by uterine artery pseudoaneurysm in hemodynamically stable patients. It underscores the value of early imaging in the detection of postoperative complications and supports consideration of nonsurgical interventions prior to surgical management when appropriate.

## Introduction

Uterine artery pseudoaneurysm (UAP) represents a rare but potentially severe vascular complication, with reported incidence rates ranging between 0.3% and 0.6% [1]. It typically results from iatrogenic or traumatic disruption of the arterial wall, most frequently associated with obstetric or gynecologic surgical procedures. In contrast to true aneurysms, which involve 3 layers of the arterial wall, pseudoaneurysms arise from a focal arterial defect allowing blood to collect outside the normal vessel lumen while remaining in communication with the parent artery, which explains its inherent instability [2].

Postpartum hemorrhage (PPH) remains one of the leading causes of maternal morbidity and mortality worldwide, requiring rapid multidisciplinary management. PPH is classified as primary when it occurs within the first day after delivery, most commonly due to uterine atony [2]. Delayed postpartum bleeding may develop several days to weeks after delivery, most commonly within the first 6 weeks, and affects a small but clinically relevant proportion of postpartum women

Secondary postpartum hemorrhage (SPPH) describes abnormal uterine bleeding occurring several days to weeks following delivery [3]. Its reported incidence varies between 0.5% and 2% of postpartum patients [4]. SPPH typically presents around 12 days postpartum, often occurring after hospital discharge, which may lead to delayed diagnosis and treatment. Common etiologies of SPPH include retained products of conception, infection, and delayed involution of the placental implantation site. Less common but clinically important causes include UAP, arteriovenous malformations, and gestational trophoblastic disease [5].

Radiologic evaluation is essential for both identifying the underlying vascular abnormality and selecting the most appropriate therapeutic approach of vascular causes of SPPH. On color Doppler ultrasound, a pseudoaneurysm (PA) is identified by the characteristic “yin-yang” flow pattern and a to-and-fro signal at its neck [6], while an associated hematoma appears as a hyperechoic retroperitoneal collection. On CT angiography, the PA is well visualized, and on unenhanced CT images, the associated hematoma demonstrates increased attenuation consistent with acute or subacute hemorrhage of approximately 54 HU.

Image-guided treatment options have become central in the management of vascular causes of SPPH, particularly UAP. Selective uterine artery embolization (UAE) is considered the first-line minimally invasive therapy in hemodynamically stable patients, as it provides rapid hemostasis while avoiding the morbidity of repeat surgery. The procedure is typically performed using a 5F uterine or visceral catheter for initial uterine artery catheterization, followed by selective navigation with a 2.0–2.4F microcatheter.

The choice of Axium Prime detachable coils combined with gelatin sponge was guided by the focal nature of the arterial injury observed on imaging. More conventional postpartum UAE materials such as PVA particles or calibrated microspheres are primarily intended for diffuse bleeding related to atony or placental bed abnormalities. In contrast, detachable coils allow precise and controllable packing of a saccular PA with a narrow neck, limiting the risk of distal migration and nontarget embolization. Gelatin sponge was added as a resorbable agent to achieve temporary flow reduction and promote thrombosis of small collateral channels, thereby reinforcing coil occlusion.

Any UAE carries a potential risk of myometrial, ovarian, or endometrial ischemia. This ischemic risk is mitigated by the use of unilateral, branch-level superselective embolization and by favoring gelatin sponge rather than permanent particle embolization of the entire uterine artery. Careful monitoring of reflux during deployment and assessment of uterine collaterals are essential to minimize nontarget ischemic complications and to preserve fertility whenever possible. This report describes a left retroperitoneal hematoma following a cesarean section caused by a UAP, highlighting the critical role of imaging in diagnosis and successful minimally invasive treatment.

## Case report

A 35-year-old woman in her third pregnancy, with a history of 2 previous cesarean deliveries and no prior viable vaginal delivery, was admitted to the obstetrics and gynecology emergency unit at 34 weeks and 3 days of gestation. Her medical history was unremarkable apart from the prior cesarean deliveries. An emergency cesarean section was performed due to complications arising during labor, which unfortunately resulted in intrauterine fetal demise.

The early postpartum period was uneventful, with no signs of life-threatening complications. However, on postpartum day 5, the patient presented to the emergency department complaining of abdominal pain, vomiting, and a general deterioration of her condition. On admission, the patient was hypotensive, with a blood pressure of 90/70 mmHg, and had no signs of external bleeding. Her hemoglobin level was 6 g/dL. She initially received 2 units of packed red blood cells, resulting in an increase in hemoglobin to approximately 7 g/dL. Subsequently, she was transfused with an additional 4 units of packed red blood cells and treated with intravenous iron sucrose (Venofer), raising her hemoglobin to 11 g/dL. Platelet count was 240 ×10⁹/L, INR 1.1, aPTT 32 seconds, and fibrinogen 3.4 g/L. Tranexamic acid 1 g intravenously was administered, and the patient received standard uterotonics consisting of oxytocin infusion 20 IU. After these measures, she became hemodynamically stable, allowing radiologic evaluation.

**Fig. 1:**
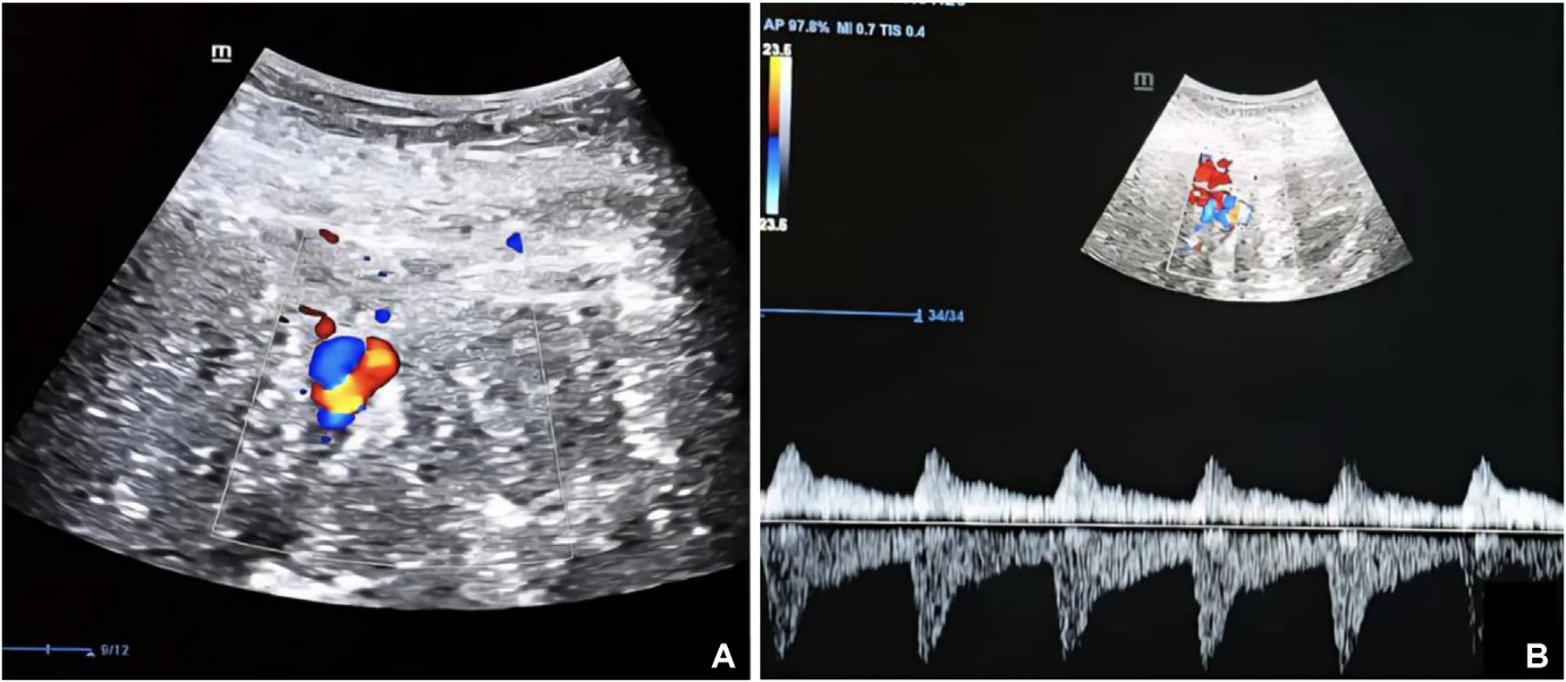
(A) Doppler Ultrasound with characteristic ``Yin Yang sign'' and (B) Pulsed wave Doppler: ``to-and-fro'' pattern refers to a characteristic bidirectional blood flow.

An emergency ultrasound with Doppler was performed, revealing a left abdominopelvic hyperechoic collection adjacent to a saccular dilatation of a vascular structure. Color Doppler imaging demonstrated bidirectional blood flow within this lesion, producing the characteristic ``yin-yang sign'' which reflects swirling blood flow typical of a PA (Fig. 1A). Pulsed wave Doppler further confirmed a ``to-and-fro'' pattern at the neck of the lesion, reflecting the classic bidirectional arterial flow seen in PA (Fig. 1B).

**Fig. 2:**
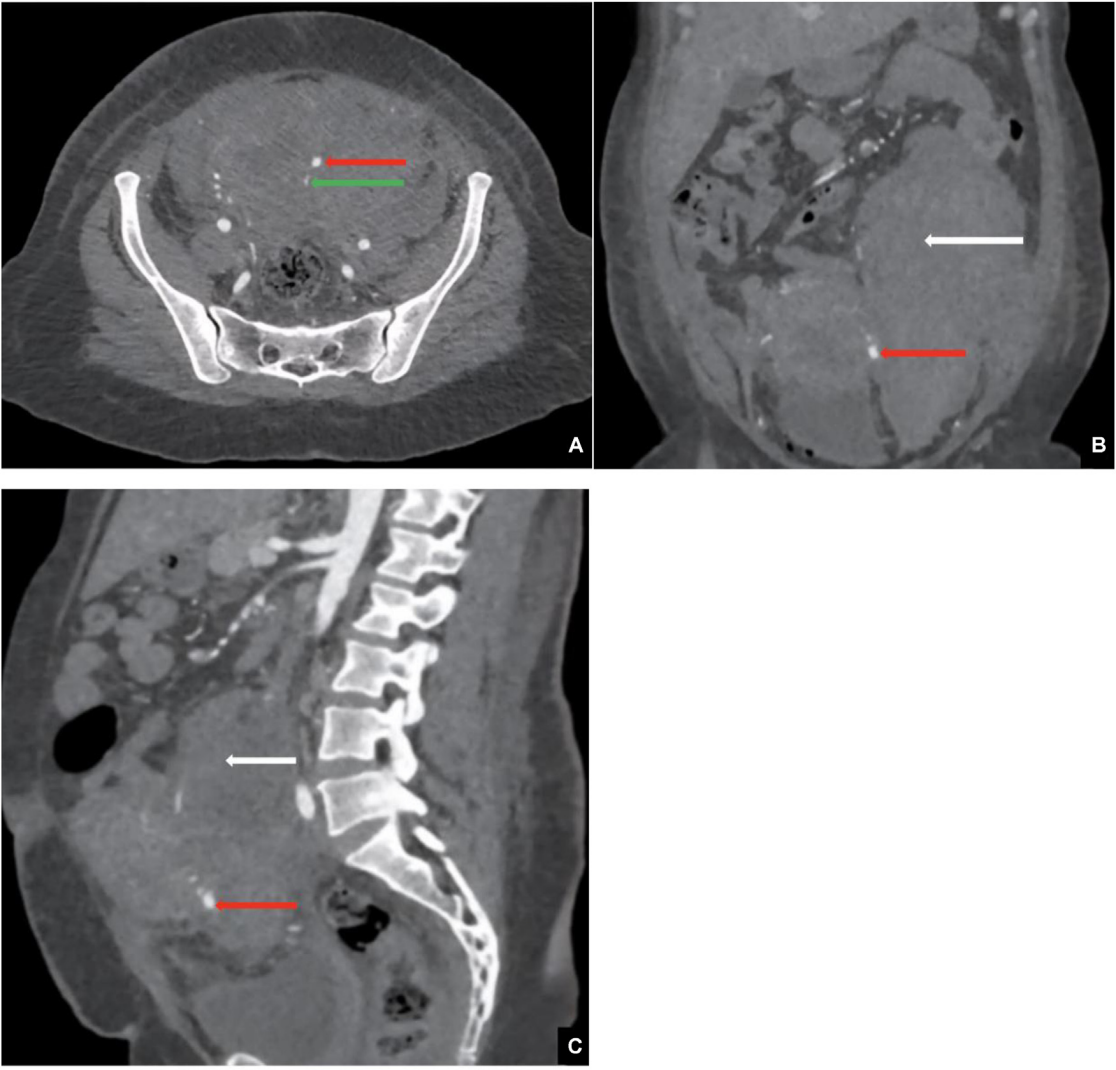
Arterial phase contrast-enhanced CT image (A) axial, (B) coronal and (C) sagittal: showing a well-defined pseudoaneurysm (red arrow) arising from the left uterine artery (green arrow), associated with a retroperitoneal hematoma (white arrow).

**Fig. 3:**
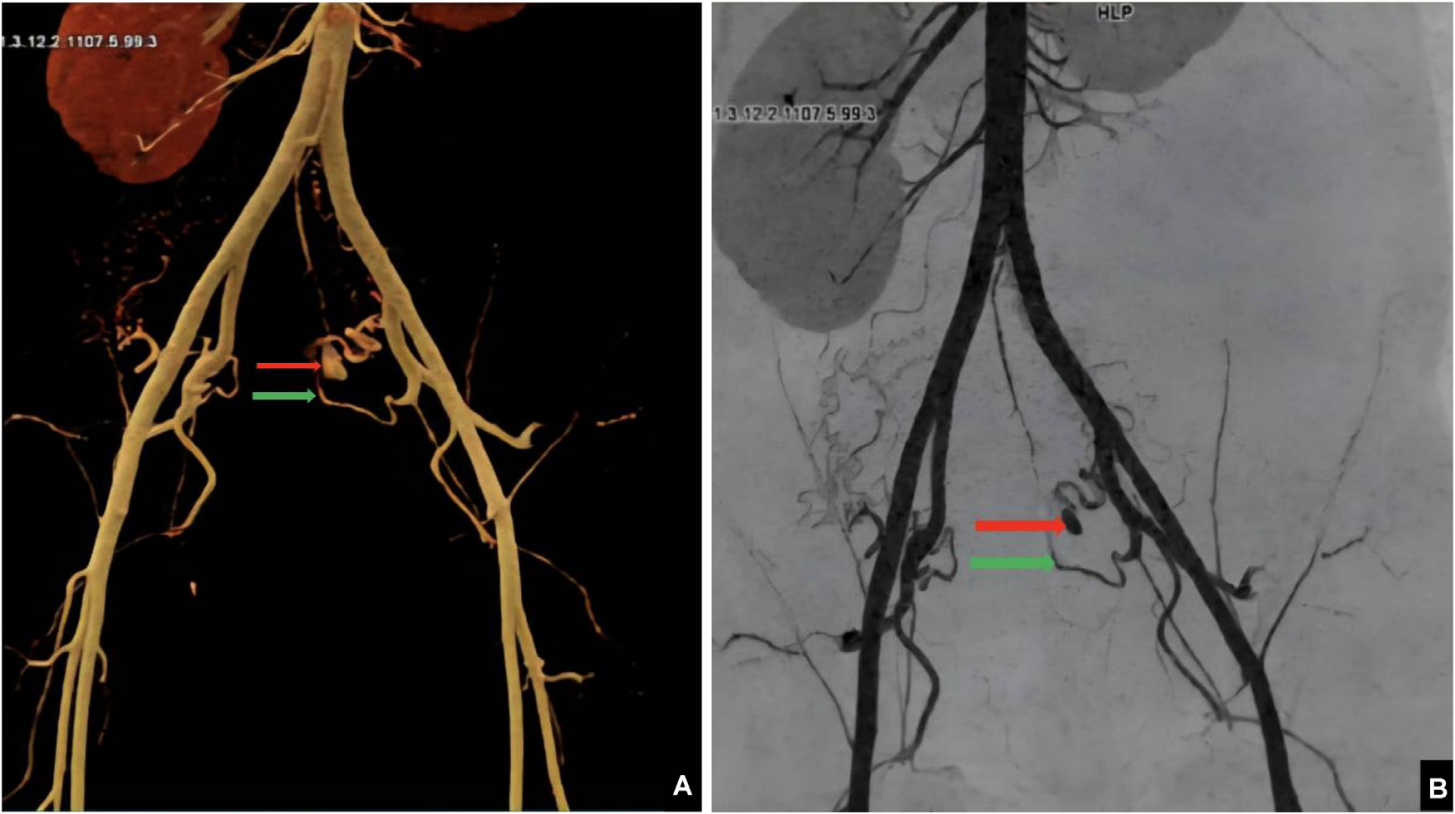
(A) Volume-rendered (VR) and (B) CT angiography image demonstrating a pseudoaneurysm (red arrow) originating from the left uterine artery (green arrow).

An abdominopelvic CT scan was performed in helical mode with multiplanar reconstruction before and after intravenous administration of 100 mL of nonionic iodinated contrast (350 mgI/mL) at a rate of 4 mL/s. Arterial phase images were acquired 25–30 seconds after injection, followed by portal venous phase imaging at 70 seconds. Noncontrast images were also obtained to assess hematoma density. The scan was performed using a GE 64-slice CT scanner. Imaging revealed a retroperitoneal hematoma measuring approximately 12.3 × 9.9 × 19 cm, appearing as a hyperdense collection of approximately 54 HU on noncontrast images. After contrast injection, a well-defined, hyperattenuating saccular lesion with smooth walls was identified adjacent to the left lateral aspect of the cervical-isthmic region of the uterus. The lesion measured approximately 4 × 5 × 8 mm (anteroposterior × transverse × craniocaudal) and was connected to a branch of the left uterine artery via a narrow neck measuring approximately 2 mm**.** It demonstrated intense contrast enhancement during the arterial phase, with no significant washout in the venous phase, confirming the diagnosis of a pseudoaneurysm of the left uterine artery (Fig. 2, Fig. 3).

After stabilizing the patient, arteriography was performed via right common femoral artery access. Selective catheterization of the left internal iliac artery was achieved using a Cobra C2 catheter advanced over a hydrophilic guidewire. Angiography confirmed the diagnosis by demonstrating a PA arising from the left uterine artery. A vasospasm of the uterine artery was noted but partially resolved spontaneously after approximately 10 minutes.

**Fig. 4:**
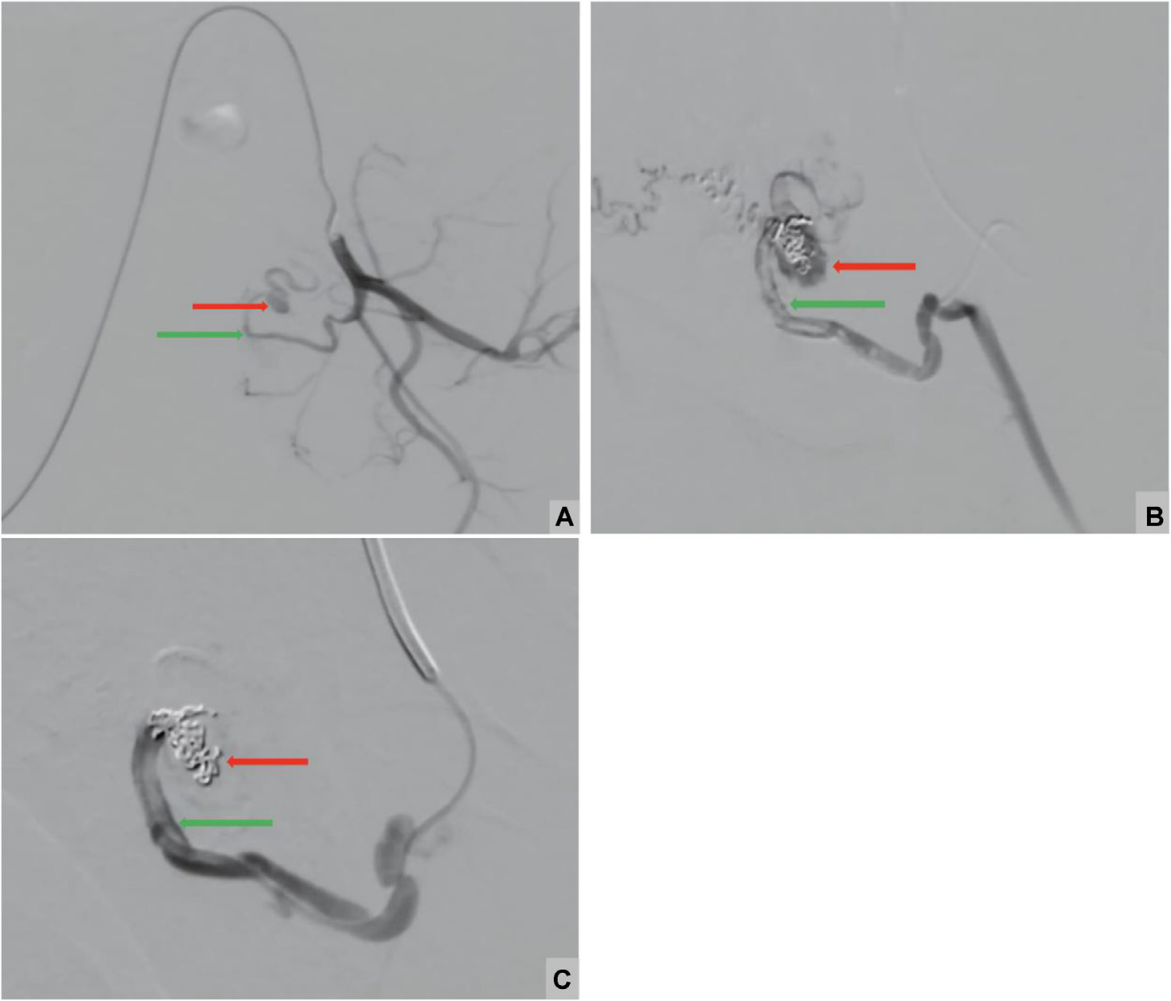
Digital subtraction angiography (DSA) images: (A) Pre-embolization: showing a pseudoaneurysm (red arrow) arising from the left uterine artery (green arrow). (B) Postembolization: showing coil deployment within the pseudoaneurysm (red arrow) arising from the left uterine artery (green arrow). Partial occlusion is noted, with residual filling of the pseudoaneurysmal sac and (C) Postembolization: complete occlusion of the left uterine artery (green artery) with no residual flow into the pseudoaneurysm (red arrow).

Catheterization of the PA proved challenging. Embolization was then performed under fluoroscopic guidance using Axium Prime detachable coils: 4 coils measuring 4 × 6 mm, 1 coil measuring 4 × 10 mm, and 1 coil measuring 5 × 15 mm. Additionally, embolization was supplemented with gelatin sponge particles fragmented to approximately 0.5 mm in size. The final control demonstrated complete occlusion of the PA (Fig. 4).

**Fig. 5:**
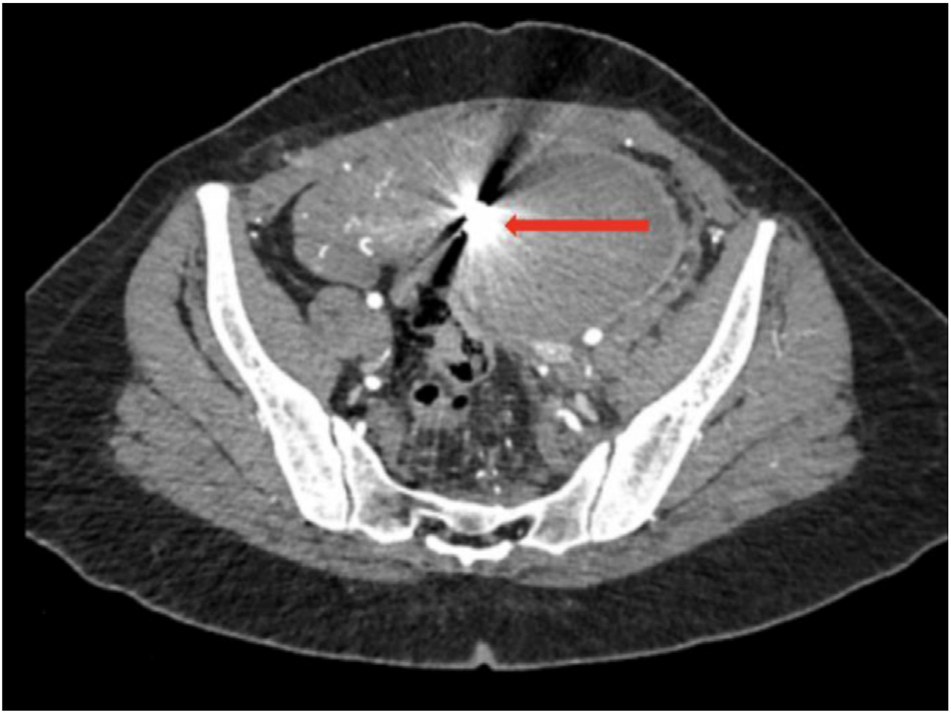
Axial arterial phase CT image showing embolization coils in place (red arrow) with complete disappearance of the previously noted pseudoaneurysm.

The patient was discharged 5 days later in stable general condition. A CT scan performed 2 weeks after discharge demonstrated complete resolution of the PA (Fig. 5) and partial regression of the retroperitoneal hematoma. Given the favorable clinical evolution, a decision was made to continue conservative management, allowing for spontaneous resolution and minimizing the risk of secondary infection that could arise from interventional evacuation.

## Discussion

PPH remains a major cause of maternal mortality worldwide. SPPH occurs days to weeks after delivery and often presents after hospital discharge, with the highest incidence typically between days 8 and 14 postpartum. While uterine atony accounts for most cases of early postpartum bleeding [7], less frequent etiologies of secondary hemorrhage include UAP, arteriovenous malformations, and choriocarcinoma [3]. When the most common causes have been excluded, diagnostic evaluation relies heavily on imaging beginning with color Doppler ultrasound to CT scan and ultimately arterial angiography, which serves both diagnostic and therapeutic purposes.

The pathophysiology of UAP involves disruption or injury to the arterial wall, most commonly following uterine trauma such as cesarean section (as in our case), dilation and curettage, myomectomy, or other pelvic surgeries. Disruption of the arterial wall allows blood to escape into the surrounding tissues while maintaining communication with the parent artery through a narrow neck. The extraluminal blood is contained by a fibrous capsule rather than the full arterial wall, forming a PA. Hemorrhage is influenced by lesion size, arterial pressure, and local tissue support, although rupture remains difficult to predict [8]. The extraluminal turbulent flow within the PA can cause progressive enlargement of the lesion, increasing the risk of rupture and severe hemorrhage.

Clinical assessment alone is often insufficient to establish the diagnosis in cases of UAP, particularly when there is no external bleeding. Patients may present with nonspecific symptoms such as deterioration of general condition, vomiting, or altered consciousness. Laboratory tests play a crucial role by detecting significant decreases in hemoglobin and platelet counts, indicative of ongoing internal hemorrhage and hemodynamic instability. Early recognition of these signs is crucial for timely diagnosis and intervention.

Imaging is essential for both diagnosis and management of UAP, beginning with noninvasive modalities. Color Doppler ultrasound is the first-line investigation, as it can identify the lesion’s origin and size, typically showing a well-defined sac with abrupt contours due to the absence of an intimal layer surrounding the PA [9]. On color Doppler, the characteristic “yin-yang” sign representing bidirectional turbulent flow coded in red and blue is observed. Pulsed wave Doppler further confirms the diagnosis by demonstrating the classic “to-and-fro” flow pattern at the neck of the lesion, reflecting the bidirectional arterial blood flow typical of PA.

Contrast-enhanced CT is a critical diagnostic tool for UAP, offering high spatial resolution and 3-dimensional volume-rendered (3D VR) reconstructions. These reconstructions typically reveal, saccular vascular lesion exhibiting contrast enhancement synchronous with the abdominal aorta during the arterial phase. The pseudoaneurysm appears as a well-defined, hyperattenuating sac with smooth walls, often arising from a branch of the uterine artery. Unlike arteriovenous malformations, this lesion maintains enhancement into the venous phase without rapid washout.

Endovascular embolization has become the preferred therapeutic approach in their management. Indeed, its advantages outweigh the rare potential drawbacks and are primarily represented by a reduction in morbidity, the ability to localize the bleeding site and provide more distal occlusion than surgical ligation, and preservation of future fertility compared to hysterectomy [3,10].

From a technical standpoint, endovascular management is usually performed through common femoral arterial access, which may be obtained on either side depending on operator preference and vascular anatomy. After insertion of a 5-French arterial sheath using standard percutaneous technique, selective angiography of the pelvic arteries is performed using a 5F diagnostic catheter (such as a Cobra C2 catheter) advanced over a hydrophilic guidewire to delineate the uterine arterial anatomy. This step allows precise identification of the PA, its arterial origin, and the exclusion of associated vascular abnormalities. Targeted catheterization of the feeding uterine artery is then achieved using a superselective microcatheter system (typically 2.0–2.4F), enabling distal and accurate access to the pathological vessel while minimizing nontarget embolization. Embolization is subsequently performed to exclude the PA from the arterial circulation, most commonly using detachable coils, with or without adjunctive embolic agents such as gelatin sponge, until complete angiographic occlusion of the lesion is confirmed. A completion angiogram is subsequently obtained, first through the microcatheter and then at a more proximal level, to verify complete exclusion of the pseudoaneurysm and to ensure that no procedure-related complications or residual abnormal flow are present.

## Conclusion

Secondary postpartum hemorrhage caused by uterine artery pseudoaneurysm is a rare but potentially life-threatening condition that requires a high index of suspicion. Early recognition using Doppler ultrasound and contrast-enhanced CT is essential for prompt diagnosis. Selective uterine artery embolization represents an effective, minimally invasive treatment that allows rapid hemorrhage control while avoiding surgical morbidity. Optimal patient outcomes depend on close multidisciplinary collaboration between obstetricians, interventional radiologists, and anesthesiologists, underscoring the importance of coordinated care in managing complex postpartum vascular complications.

## Patient consent

Complete written informed consent was obtained from the patient for the publication of this study and accompanying images.
